# STCRDab: the structural T-cell receptor database

**DOI:** 10.1093/nar/gkx971

**Published:** 2017-10-26

**Authors:** Jinwoo Leem, Saulo H P de Oliveira, Konrad Krawczyk, Charlotte M Deane

**Affiliations:** Department of Statistics, University of Oxford, 24–29 St Giles, Oxford, OX1 3LB, UK

## Abstract

The Structural T–cell Receptor Database (STCRDab; http://opig.stats.ox.ac.uk/webapps/stcrdab) is an online resource that automatically collects and curates TCR structural data from the Protein Data Bank. For each entry, the database provides annotations, such as the α/β or γ/δ chain pairings, major histocompatibility complex details, and where available, antigen binding affinities. In addition, the orientation between the variable domains and the canonical forms of the complementarity-determining region loops are also provided. Users can select, view, and download individual or bulk sets of structures based on these criteria. Where available, STCRDab also finds antibody structures that are similar to TCRs, helping users explore the relationship between TCRs and antibodies.

## INTRODUCTION

T-cell receptors (TCRs) are proteins of the adaptive immune response. They are expressed on the surfaces of T-cells and typically recognise peptides that are presented by major histocompatibility complex (MHC) molecules. Despite their micromolar binding affinity and potential cross-reactivity, TCRs are selective for foreign peptide-MHC complexes on antigen presenting cells (APCs; 1–3). Upon binding, TCRs can activate the T-cell for direct killing of APCs, or stimulate other components of the adaptive immune system, such as B-cells (4–6). The clinical relevance of TCRs has attracted interest in understanding the structural basis of a TCR’s activity (7,8), and exploring the possibility of designing TCRs as novel biotherapeutics (9).

Given the sensitivity of TCR-MHC interactions and the extreme diversity of the TCR repertoire (10,11), computational methods are increasingly being used for rational TCR design (10,12–15). TCR structural data is an invaluable resource for designing and developing computational tools, for example, template-based modelling pipelines (12).

A small number of publicly available databases focus on delivering TCR-specific data (16–18). McPAS-TCR (18) is a manually curated database that maps αβ TCR sequences to pathogens or epitopes (18). The database does not contain structural information, making it difficult to determine the importance of specific residues in MHC and antigen binding. There are two databases that contain some TCR structural information: ATLAS (16) and IMGT (17). ATLAS is a manually curated database, containing a large volume of affinity data; users can view and download one of 87 experimental structures, and retrieve summaries of individual queries. The bulk of the structural data in ATLAS is comprised of homology models of variants of experimental structures. These structures lack annotations that can be useful for further analyses (e.g. numbering; 16). Once again like McPAS-TCR, only αβ TCRs are annotated. IMGT (17) has a richer (308 experimental structures) and more diverse set of structural data (e.g. γδ TCRs). However, it is only possible to search based on a limited set of attributes; for example, it is not possible to specify the peptide sequence of the antigen. In addition, IMGT does not allow users to generate bespoke datasets for analysis (17).

We have developed the Structural TCR Database (STCRDab), building on our Structural Antibody Database (SAbDab; 19). STCRDab is a TCR database that automatically collects and curates data on a weekly basis. Users can browse and select both αβ and γδ TCRs based on a wide range of criteria, such as the sequence of the TCR’s complementarity-determining region (CDR) loops, the resolution of the structure, and the type of MHC molecule bound by the TCR. Users can also search by structural annotations, such as the orientation between the TCR’s variable domains (20). STCRDab is linked to SAbDab, so that users can find antibody structures that are similar to TCRs, providing insight into designing TCR-like antibodies and chimaeric antigen receptors. Following a query, users can inspect and download individual or sets of TCR structures. Each search generates a unique zip file, containing a summary of the search and Protein Data Bank (PDB) format files of structures that match the query (21,22).

### Structure nomenclature

**Figure 1.**
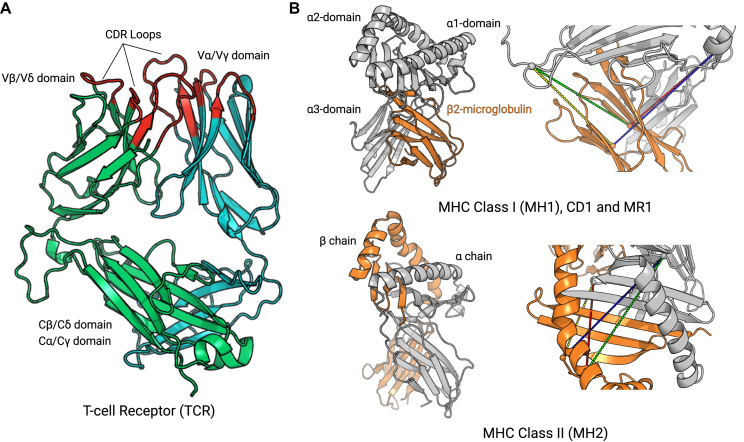
Nomenclature and colouring scheme used in STCRab. (**A**) T-cell receptors (TCRs) are formed from two chains: TCRβ/TCRα (to form αβ TCRs, as shown), or TCRδ/TCRγ (to form γδ TCRs). The residues coloured in red indicate the IMGT–defined CDR loops. This colouring scheme is also used on the website. (**B**) Major histocompatibility complex (MHC) molecules can be divided into classical and nonclassical MHCs. MH1 and MH2 are considered ‘classical’ MHCs, while CD1 and MR1 are ‘nonclassical’. However, CD1 and MR1 are structurally similar to MH1, whereas MH2 is structurally distinct. To pair MH1, we use the following distance constraints: α15–β23 (green; 32 Å), α15–β104 (yellow; 32 Å), α51–β23 (red; 32 Å), α51–β104 (blue; 37 Å). To pair MH2, the following distance constraints are used: α29–β64 (green; 34 Å), α29–β39 (yellow; 22 Å), α37–β64 (red; 32 Å), α37–β39 (blue; 28 Å).

STCRDab is primarily focussed on consistently annotating TCR structural data, but also numbers MHC molecules consistently. The terminology for both types of structures is shown in Figure 1 and described below.

#### TCR structures

The majority of available TCR structures are αβ TCRs, which are formed of TCRα and TCRβ chains. A small number of TCRs are γδ TCRs, consisting of TCRγ and TCRδ chains. The TCRβ and TCRδ chains are considered to be analogous to antibody heavy chains while the TCRα and TCRγ chains are considered to be analogous to antibody light chains (23).

Each TCR chain is characterised by two immunoglobulin domains: a variable domain (*V*) and a constant (*C*). Both variable and constant domains have a conserved β–sandwich structure (Figure 1), making it possible to number and compare variable domains from different TCRs (24). In STCRDab, we use the IMGT numbering as it provides consistent numbering for the CDR loops (21), and has been used on other occasions for structural analysis of TCRs (8,20). On each variable domain, there are three hypervariable loops that have the highest degree of sequence and structural variation, known as the CDRs. Flanking the CDRs, the remaining portions of the TCR structure are collectively known as the TCR’s ‘framework’.

#### MHC structures

APCs use either the ‘classical’ MHC to present peptide antigens, or the ‘nonclassical’ MHC–like molecules to present lipid molecules or vitamin B precursors (25). The classical MHCs can be subdivided into MHC class I (MH1) and MHC class II (MH2), while the nonclassical MHC–like molecules include cluster of differentiation 1 (CD1) and MHC class I–related protein (MR1). Both classical and nonclassical MHCs have an antigen binding groove formed by a β-sheet, flanked by two α helices (Figure 1). MH1, CD1 and MR1 are formed by the pairing of the MHC chain and a β_2_ microglobulin, while MH2 is formed by the MHCα and MHCβ chains. As with the TCR structures, the IMGT numbering is used for MHCs (22).

## DATA SOURCES AND CONTENTS

### TCR structures

**Figure 2.**
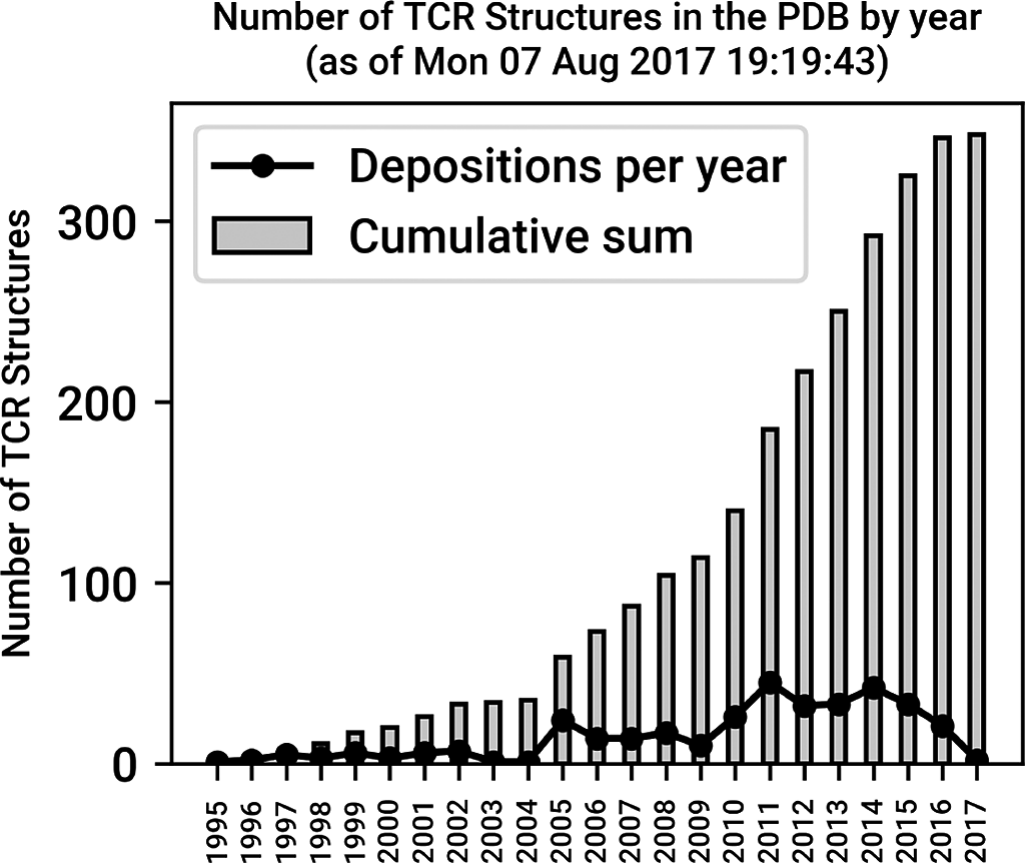
Growth of TCR structures in the PDB. As of 7 August 2017, there are 348 entries of human and mouse TCR structures in the PDB. On average, two new structures have been deposited per month since 2007.

**Figure 3.**
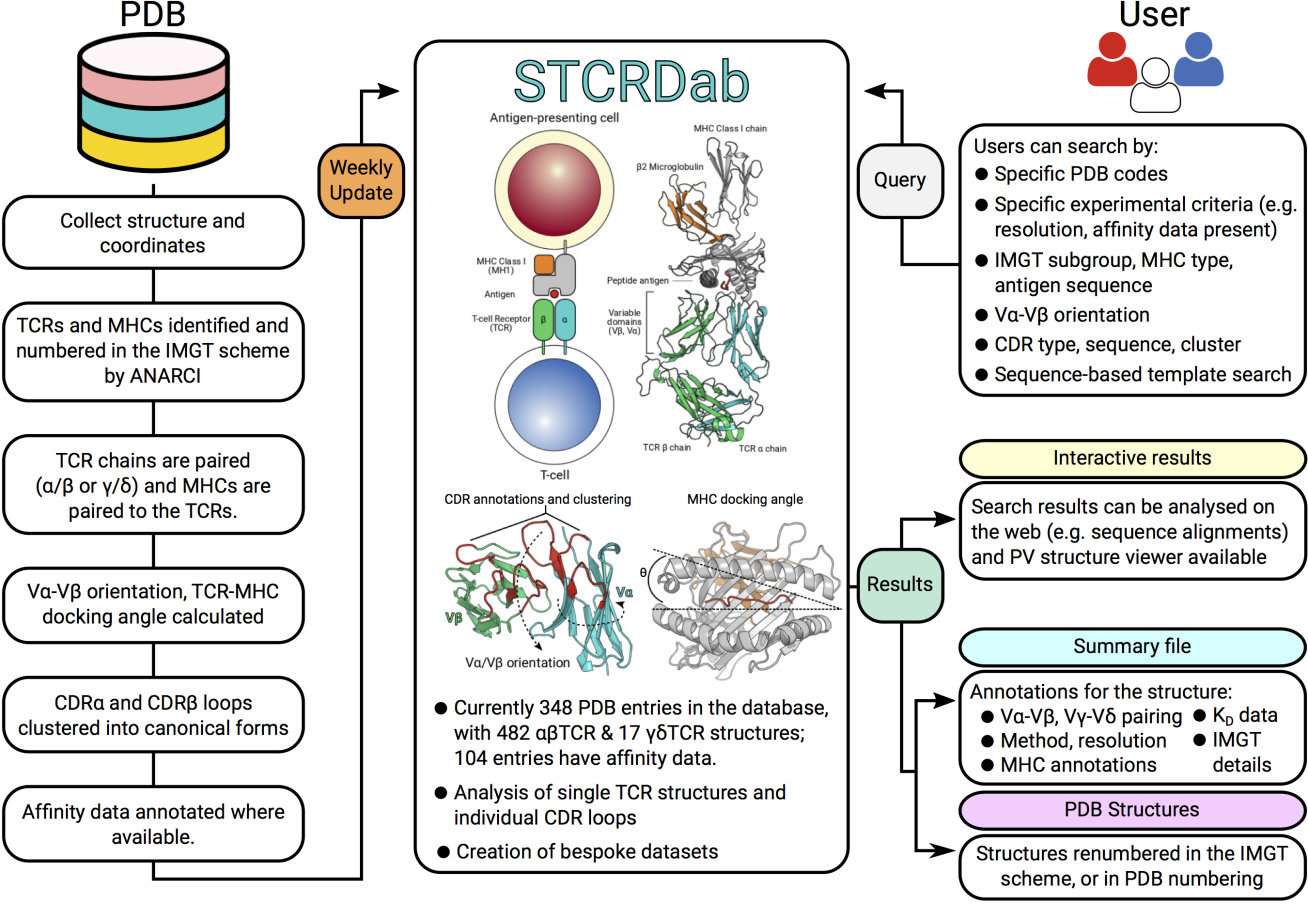
Workflow for STCRDab. Every week, STCRDab automatically detects and numbers newly released TCR structures from the PDB using ANARCI (24). Any MHC or MHC-like molecules are also numbered by ANARCI. Each structure is automatically annotated with several structural properties, such as its TCR-MHC docking angle (23). Users can submit a variety of queries to STCRDab to retrieve structures. Users are given their results for online analysis, and custom datasets are dynamically generated for download.

As of 7 August 2017, STCRDab contains 348 entries with at least one TCR chain. On average, two TCR structures have been deposited in the PDB per month since 2007 (Figure 2). STCRDab is automatically updated weekly, in line with the PDB updating schedule (Figure 3). Paired αβ TCRs form the majority of the data, followed by single TCR chains, e.g. Vβ only structures, then γδ TCRs (Table 1). There are also structures that fit none of these categories – for instance, an engineered TCRδ/TCRα receptor (PDB: 4wo4).

**Table 1.** Number of TCR structures in STCRDab

TCR type	Unbound	Classical MHC	Nonclassical MHC	Total
αβ TCR	91	273	118	482
γδ TCR	12	0	5	17
Unpaired TCR	80	0	0	80
Other	0	7	2	9

An entry in STCRDab can have multiple TCR structures, e.g. PDB: 2vlr.

STCRDab uses a modified version of ANARCI (24) to detect and number any human and mouse TCR and MHC sequences in newly–released structures from the PDB. Briefly, ANARCI aligns sequences to a database of hidden Markov models (HMMs). The HMMs were built using multiple sequence alignments of human and mouse TCR and MHC sequences from IMGT (17). We only pursue further annotation if there is at least one TCR chain in the PDB entry.

Similar cutoffs to those in SAbDab (19) are used for pairing αβ TCRs and γδ TCRs. We consider two TCR chains to pair if the distance between the Cα atoms of the conserved cysteines at IMGT positions 104 in each domain is <22Å. MHC chains are paired by using four distance constraints (Figure 1). For any MHC molecule that pairs with a β_2_ microglobulin, i.e. MH1, CD1, and MR1, we calculate the distance between the Cα atoms of IMGT positions 15, 51 on the MH1/CD1/MR1 chain, and the Cα atoms of IMGT positions 23 and 104 in the β_2_ microglobulin. For MH2 molecules, the distances between the Cα atoms of IMGT positions 29 and 37 in the α chain and IMGT positions 39 and 64 in the β chain are used.

The paired TCR and MHC molecules are matched together if there is at least one Cβ atom from the TCR’s CDR3 loops that is within 8Å of any of the Cβ atoms in the helix regions of the MHC (8). The putative TCR-MHC pair with the highest number of Cβ-Cβ contacts is set as the TCR-MHC complex. Potential antigens are then identified by searching for proteins, peptides, and other non-polymeric ligands (haptens). Peptide and protein antigens are matched if a Cβ atom is within 8Å of the TCR-MHC complex. For haptens, we apply a 3.5Å cutoff between its atoms and the MHC, and an 8Å cutoff with Cβ atoms of the TCR’s CDR3 loops. Unconventional structures, such as PDB: 2icw (which features a protein that is between the MHC and TCR), are flagged for manual inspection.

### Vα-Vβ orientation, docking angle

In order to describe the TCR binding mode with the MHC, we use a TCR-specific version of ABangle (TRangle; 20), and calculate the docking angle between the TCR and the MHC (23). TRangle describes the relative orientation between the Vα and Vβ domains using six parameters. The effect of Vα-Vβ orientation on MHC binding is not yet clear, though it can provide the basis for engineering TCR-like antibodies, or antibody-like TCRs (20). STCRDab automatically calculates the TRangles for αβ TCRs. Due to the small amount of data, the TRangle method is currently not used for γδ TCRs; however, as data increases, this will become possible.

The docking angle describes how the TCR engages with the MHC. Here, we implement a previously established formula to calculate the docking angle (23).

### Complementarity-determining region loops and clustering

In STCRDab, the CDR loops are identified using the IMGT definition (21): CDR1 (IMGT 27–38), CDR2 (IMGT 56–65) and CDR3 (IMGT 105–117). The CDRα1, CDRα2, CDRα3, CDRβ1, CDRβ2 and CDRβ3 loops have been clustered into canonical forms (12,26), as has been done for the CDR loops of antibody structures (27–29).

We have clustered the CDR loops of TCRs using a length-independent density-based clustering (DBSCAN) method, as first proposed for antibodies (29). Briefly, we took the CDR loops of all TCRβ and TCRα structures with resolution ≤2.8Å; we removed loops with missing residues, or those that have at least one backbone atom with a *B*-factor of 80 or higher. We then calculated the length-independent root-mean square deviation (RMSD) between CDR loops using a dynamic time warp algorithm. The RMSD matrix is then clustered using DBSCAN. To compare our newly identified clusters, we map them to canonical forms from previous studies (12,26). The canonical forms for the CDRα3 and CDRβ3 loops are currently early-stage observations and are not yet useful for modelling (12). However with more data, we should have more accurate definitions of the canonical forms in TCRs, as is the case in antibodies.

### TCR binding affinity

The binding affinities of TCR-MHC complexes were manually curated from PDBBind (30) and ATLAS (16). Where possible, experimental details describing how the affinity was measured (e.g. surface plasmon resonance) were also annotated. For cases where the affinity of a TCR-MHC complex was measured in multiple studies (e.g. PDB: 3qdj), the values from the authors that determined the TCR structure are cited. There are currently 104 entries in STCRDab with a *K*_D_ value. These values should serve as a useful resource for those interested in TCR docking and design.

## DATA ACCESS

### Download options

STCRDab provides a tab-separated file that summarises the results of a particular query with annotations for each TCR structure. STCRDab also provides two sets of structure files: either the raw file directly from the PDB (31), or a structure file in re-numbered in the IMGT scheme (21,22) via ANARCI (24).

In the re–numbered PDB structure file, TCR variable domains, MHC G-domains, and the β2 microglobulin are numbered in the IMGT scheme (21,22). All non-TCR and non-MHC chains retain their numbering as in the original PDB file. The header of the renumbered PDB file contains TCR pairing information, along with their paired MHC and antigen in a REMARK field. For instance, in the entry 2vlr, there are two TCR structures, formed between chains E and D, and between chains J and I. The TCR E-D binds to the peptide antigen (chain C) presented by the MH1 molecule (A-B); likewise, TCR J-I binds to the antigen on chain H, presented by F-G. Thus, the header shows


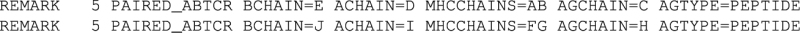


The tab-separated summary file contains more detailed information about each entry, and can be used by most spreadsheet applications. Each column of the tab-separated file contains more information for the TCR, such as the pairing information, and the paired MHC type.


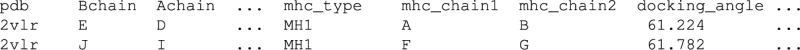


The summary file is highly flexible as it can be generated for one particular entry (as shown), or for a collection of entries that satisfy a user’s search criteria. For every search, users can download these files individually per entry, or as a zip file that contains the collection of PDB files and the summary file.

### Analysis of individual structures

**Figure 4.**
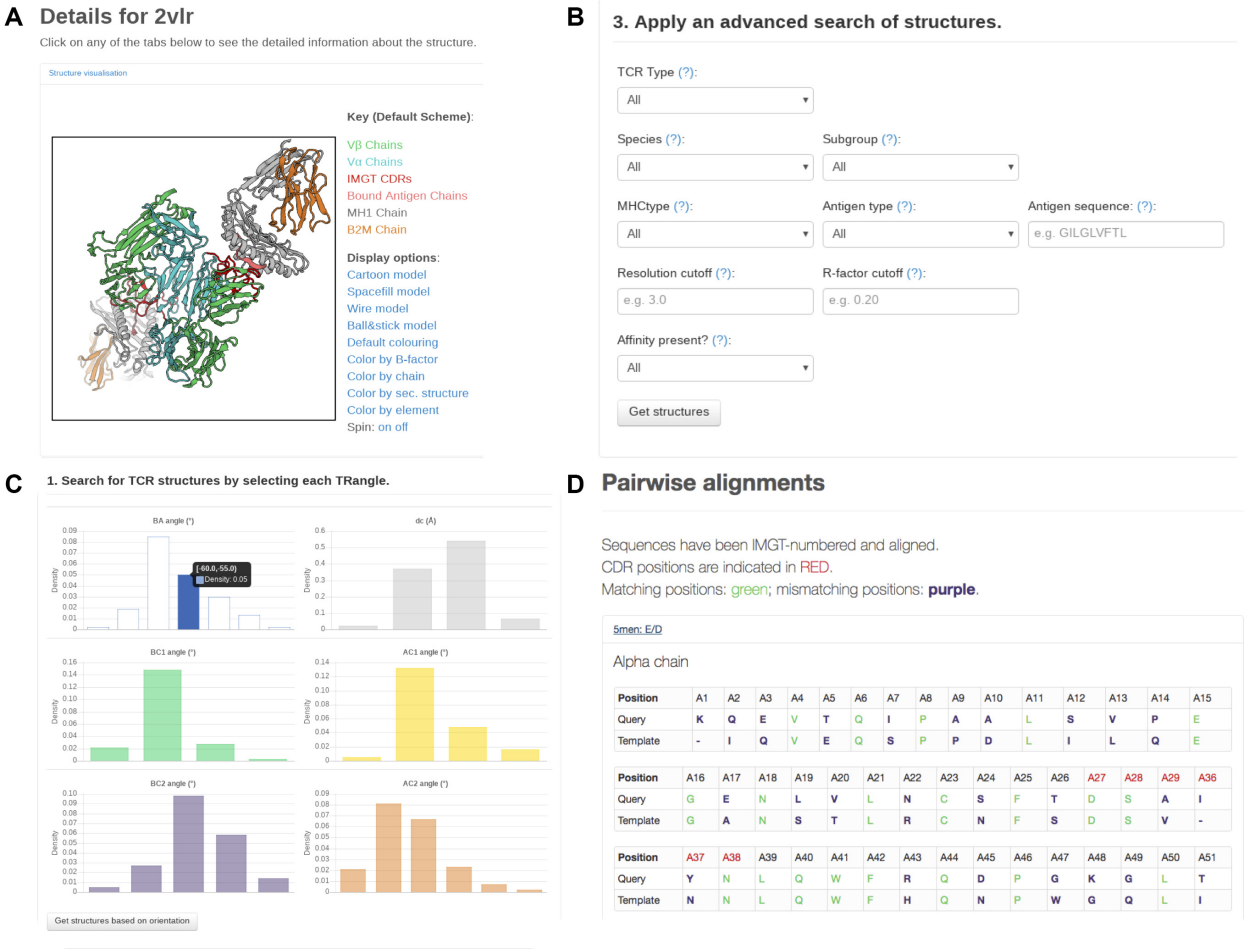
Screenshots of STCRDab. (**A**) Summary page for a single TCR structure in STCRDab. Each page has the IMGT-numbered TCR structure visualised in PV (32), followed by additional details and links. (**B**) The advanced ‘TCR search’ page gives users the option to specify a range of criteria to retrieve a subset of structures from STCRDab. (**C**) The orientation search page allows users to retrieve a subset of structures based on the TRangle parameters. (**D**) The sequence search results page shows how the query sequence is aligned with those in STCRDab.

An individual entry can be viewed interactively using its PDB accession code (e.g. 2vlr). Users will be directed to the summary page as shown in Figure 4A. The structure is visualised by BioPV (32); by default, the colouring scheme from Figure 1 is applied. It is also possible to use different representations (e.g. ball-and-stick model) and colouring schemes (e.g. colour by *B*-factor).

Below the viewer, STCRDab shows details of the PDB entry, including the organism information (as listed in the PDB), the method for structure determination, and the method used for measuring the affinity. Next, for each detected TCR in the PDB entry, STCRDab provides additional annotations, such as the IMGT subgroup, the species of the IMGT subgroup, and the IMGT-numbered sequence, along with a FASTA file for the TCR sequence. Where relevant, STCRDab provides the orientation, docking angles, and links to view individual CDR loops.

At the end of the summary page, users are given links to download PDB files, either raw or re-numbered, along with the summary file.

### Analysis of individual CDR loops

Each CDR loop has a unique CDR viewer page, which is both loop and chain-specific (e.g. CDRβ3 loop on chain E). STCRDab assigns the canonical class for the CDR loop where possible. In addition, if the CDR loop has a length-matched backbone RMSD of less than 1.5Å to an analogous antibody CDR loop (i.e. CDRα1/CDRL1), STCRDab provides links to antibody structures from SAbDab (19).

### Search options

Users can browse and search for TCR structures using a variety of options. For every query, a table is dynamically generated, summarising the results and providing links to summary pages and downloads.

#### Advanced search

The advanced search tool can help users filter TCR structures by TCR type (e.g. αβ TCR/γδ TCR), the IMGT species (human or mouse) and subgroup (e.g. TRAV10) of the TCR. It is also possible to apply filters based on the MHC type, or the antigen’s type (e.g. peptide versus hapten) and sequence. Finally, users can select structures based on quality (resolution, *R*-factor), and if there is affinity data available for the TCR. Upon submission, users will be given a table listing the TCR structures that satisfy the specified criteria.

#### CDR Search

The CDR search engine provides similar criteria to the advanced search tool, such as antigen type, MHC type, and defining the IMGT subgroup of the TCR. Additional attributes, such as the type of CDR, loop length and canonical class are also available. Users are provided with a table showing the PDB codes and CDR sequences of every hit. The sequences are hyperlinked to their respective entries. The data can be downloaded as described in the ‘Download options’ section above.

#### Orientation search

The orientation search tool allows users to select TCRs by choosing specific bins of TRangle parameters from the interactive online graphs. It is not necessary to choose the bins for all six parameters, as unselected parameters act as a ‘wild card’. Users can also search for antibodies based on a particular TCR structure in a specific entry, e.g. 2vlr:ED. Since orientation calculations are only performed for αβ TCRs, this search tool will only retrieve αβ TCR structures.

Following selection, STCRDab returns a list of TCR structures that are within the user-defined Vα-Vβ orientation space. Where possible, an antibody with a similar orientation to a TCR structure is also retrieved from SAbDab (19). To define orientation similarity, we use the ABangle distance measure *d*_ABangle_, defined as

(1) \begin{equation*} d_{\textrm {ABangle}} = \sqrt{ \sum ( \theta _{i,a} - \theta _{i,t} )^2 }. \end{equation*}

*d*_ABangle_ represents the Euclidean distance between the *i*th ABangle parameter, θ_*i*,*a*_ and its analogous TRangle parameter θ_*i*,*t*_, e.g. ‘HL’ angle of antibodies vs. the ‘BA’ angle of TCRs. A TCR structure *t* and an antibody structure *a* were considered to be similar if *d*_ABangle_ is less than or equal to 10. We only list the closest antibody structure, i.e. lowest *d*_ABangle_ in the orientation search results page.

#### Sequence search

The sequence search engine allows users to submit TCR sequences and retrieve TCR structures that can be used as templates for template-based modelling tools. Users can submit the sequences of the TCRβ and/or TCRα chains to find templates based on sequence identity across the entire variable domain, the framework, or the CDR loops. STCRDab uses ANARCI to detect and number variable domains in the query sequence. STCRDab returns *N* structures in decreasing order of matched sequence identity. Given the number of structures that are available, this search method is only enabled for TCRβ and TCRα chains.

## CONCLUSION

STCRDab automatically collects and curates TCR structural data from the PDB. STCRDab builds upon the foundations of our antibody database, SAbDab, in order to provide consistent annotations, and open a gateway for users to easily access, view, and download custom datasets for analysis. The database aims to act as a resource for the emerging field of computational TCR design, and to help uncover the unique structural properties of TCRs. STCRDab also provides a bridge to the extensive knowledge base of antibody structures in SAbDab, which can potentially be used to inform TCR-like antibody design or antibody-like TCR design. The database is entirely open-access and available at http://opig.stats.ox.ac.uk/webapps/stcrdab.
